# Total Joint Arthroplasty Complication and Revision Surgery Rates Vary by Region and Season: Analysis of a Large Nationwide Database

**DOI:** 10.5435/JAAOSGlobal-D-22-00109

**Published:** 2023-01-10

**Authors:** Ankur Khanna, Alex A. Betech, Andrew G. Chapple, Peter C. Krause, Vinod Dasa

**Affiliations:** School of Medicine, Louisiana State University Health Sciences Center, New Orleans, LA (Mr. Khanna); Department of Orthopedic Surgery (Dr. Betech, Dr. Krause, and Dr. Dasa) and the Department of Biostatistics (Dr. Chapple), Louisiana State University Health Sciences Center, New Orleans, LA.

## Abstract

Total joint arthroplasty (TJA) is a highly successful surgical intervention, but a subset of patients will experience postoperative complications—with some cases even needing a revision surgery. This study investigated the effects of region and season on 90-day complication rates and 1-year revision surgery rates for TJA patients.

**Methods:**

We queried the American Academy of Orthopaedic Surgeons American Joint Replacement Registry for primary TJA conducted between 2018 and 2020. Multivariable logistic regression was conducted to investigate the effects of region, season, or their interaction on the risk of complications within 90 days and the risk of revision surgery within 1 year after adjusting for race, surgery year, age group, procedure, and Charlson Comorbidity Index score. Unmeasured variables including hospital volume and surgeon ability were controlled for as nested random effects in the model. Bonferroni-adjusted LSMeans were used to compare each season, region, and season within each region.

**Results:**

The risk of complications in the West was significantly higher than in the Northeast (aOR = 2.76, *P* < 0.001), Midwest (aOR = 2.44, *P* < 0.001), or South (aOR = 3.33, *P* < 0.001). The West also had a significantly higher risk of revision surgery than the South (aOR = 1.27, *P* = 0.038). These trends held across each season. Fall procedures had a significantly lower risk of both complication and revision surgery than those in the summer (aOR = 0.85, *P* < 0.001; aOR = 0.77, *P* < 0.001) or winter (aOR = 0.89, *P* = 0.024; aOR = 0.73, *P* < 0.001). Analysis of regional-seasonal interaction found that within the Northeast, fall surgeries had a significantly lower risk of revision surgery than spring surgeries (aOR = 0.64, *P* = 0.003).

**Conclusion:**

Our study found a statistically significant increase in the risk of complication in the West compared with the Northeast, Midwest, and South. Patients in the South also had a demonstrably lower risk of revision surgery than those in the West. Seasonally, fall TJA operations demonstrated a lower risk of both complication and revision surgery compared with summer or winter operations.

Total joint arthroplasty (TJA) remains a highly successful surgical intervention, and its utilization is only projected to grow in the coming years. Current estimates suggest that by 2030, there will be a 71% and 85% increase in the annual volumes of primary total hip arthroplasty (THA) and total knee arthroplasty (TKA), respectively.^1^ This demand for TJA has placed a strain on the healthcare system in the United States and has brought about a renewed focus on ways in which to characterize and mitigate this burden.^2^

Despite the success of TKA and THA, a subset of patients will experience complications within 90 days of their index TJA. In fact, studies show that up to 16% of patients who undergo TJA will face complications in the postoperative period.^3^ A further subset of these patients will face more suboptimal outcomes and will require revision surgery within 1 year of the primary procedure, increasing the load on the healthcare system. There are several reasons why revision surgery might be required including infection, aseptic loosening, stiffness, patellar clunk, wound healing problems, and other postoperative complications.^4^ Although these causes have been investigated at some length in the literature, no study has focused on complication and revision surgery rates according to region and season.

Previous studies on other surgical interventions have found differences in complication and revision surgery rates when stratifying for patient cohorts by region and season,^5‐7^ but literature specific to TJA has concentrated on documenting only seasonal variations in the potential causes of revision surgery, omitting larger reviews of the complication and revision surgery rates themselves. The conclusions drawn from those studies have also been mixed. Anthony et al^8^ found that the risk for surgical site infections (SSIs) after TJA was highest in the summer. Postoperative complications in TKA have also been demonstrated to peak in the summer months.^9^ Yet, another study found that there was no consistent seasonal variation in the risk of revision for periprosthetic joint infection after TJA.^10^ Thus, the primary objective of this study was to investigate the effects of region and seasonality on the 90-day complication rates and 1-year revision surgery rates for primary TJA patients.

## Methods

Data for this study were obtained by querying the AAOS American Joint Replacement Registry (AJRR) for all patients who underwent a primary THA or TKA between 2018 and 2020. The AJRR is a nationwide database which includes procedural, postoperative, and patient-reported outcome measures data from hospitals, ambulatory surgical centers, and private practice groups across the United States. This includes geographic information on each procedure, dividing the US along the four census regions: Northeast, Midwest, South, and West.

We performed a retrospective cohort analysis examining the relationship between the risk of complications and the region and season of the index TJA. Our outcome of interest for the analysis was whether a patient had complications within the 90 days after their initial operation. We performed a second retrospective cohort analysis to determine whether there was a relationship between the risk of revision surgery and the region or season of the index procedure. Our outcome of interest for this second analysis was whether a patient had a revision surgery within 1 year of their original surgery. Reasons for complications and revision surgery submitted to the AJRR included SSI/inflammation, pain, hematoma/wound complications, instability, aseptic loosening, fracture, and mechanical complications.

Other variables collected included age, race, sex, Charlson Comorbidity Index (CCI) score, procedure type (THA or TKA), procedure year, season, and geographic region. Categorical covariates were summarized by region by reporting the count and percentage, and continuous covariates were summarized by reporting the mean with the standard deviation. *P* values were not reported for unadjusted comparisons because they depended on the procedure year, hospital where the surgery took place, and surgeon identification.

Multivariable logistic regression adjusting for age, race, CCI score, procedure, and year was used to assess the effects of region, season, or their interaction on the risk of complication and revision surgery. Nonmeasured factors such as hospital or surgeon ability were controlled for as nested random effects. Additional analysis was conducted comparing each region, season, and region within each season to determine relative effects. Bonferroni corrections were applied to the LSmeans for these comparisons due to the increased risk of type I error when implementing multiple between-group comparisons. All statistical analyses were performed using SAS (version 9.0; SAS Institute). Significance was set at 0.05. All plots were made using R statistical software (version 4.0.1; The R Foundation).

## Results

Our study included 714,530 primary TJA patients. Most underwent surgery in the Midwest (28.57%) and the South (28.94%), a trend that remained consistent across all four seasons. There were 416,448 (58.28%) female patients. Among all included patients, 418,263 (56.26%) underwent TKA. The South had the most African American patients (49.44% of total African American patients), whereas the West had the most Asian patients (49.17% of total Asian patients). The West also had the highest proportion of patients who identified with two or more races (53.5% of total multirace patients). A total of 7147 patients (1.00%) experienced complications within 90 days of their index TJA, and 5071 total patients (0.71%) underwent a revision surgery within 1 year of their initial surgery. Additional demographic data within each considered region are presented in Table 1.

**Table 1:** Baseline Patient Characteristics by Geographic Region^a^

	Northeast (N = 137,259)	Midwest (N = 204,109)	South (N = 206,757)	West (N = 166,405)	Total (N = 714,530)
Season					
Winter	36,819 (26.82%)	51,663 (25.31%)	54,292 (26.26%)	43,424 (26.10%)	186,198 (26.06%)
Spring	31,303 (22.81%)	47,444 (23.24%)	52,639 (25.46%)	43,956 (26.42%)	175,342 (24.54%)
Summer	34,783 (25.34%)	52,760 (25.85%)	51,553 (24.93%)	40,771 (24.50%)	179,867 (25.17%)
Fall	34,354 (25.03%)	52,242 (25.60%)	48,273 (23.35%)	38,254 (22.99%)	173,123 (24.23%)
Race					
Missing	14,588 (10.63%)	13,499 (6.61%)	20,878 (10.10%)	18,338 (11.02%)	67,303 (9.42%)
American Indian	2,513 (1.83%)	2,150 (1.05%)	846 (0.41%)	3,226 (1.94%)	8,735 (1.22%)
Asian	1,961 (1.43%)	1,171 (0.57%)	1,733 (0.84%)	4,707 (2.83%)	9,572 (1.34%)
Black/African American	9,676 (7.05%)	8,805 (4.31%)	22,036 (10.66%)	4,058 (2.44%)	44,575 (6.24%)
Native Hawaiian/Other Pacific Islander	68 (0.05%)	59 (0.03%)	73 (0.04%)	564 (0.34%)	764 (0.11%)
White	106,570 (77.64%)	177,701 (87.06%)	160,406 (77.58%)	131,606 (79.09%)	576,283 (80.65%)
Two or more	1,883 (1.37%)	724 (0.35%)	785 (0.38%)	3,906 (2.35%)	7,298 (1.02%)
Sex					
Missing	8 (0.01%)	5,482 (2.69%)	37 (0.02%)	211 (0.13%)	5,738 (0.80%)
Female	81,173 (59.14%)	116,426 (57.04%)	121,811 (58.92%)	97,038 (58.31%)	416,448 (58.28%)
Male	56,078 (40.86%)	82,201 (40.27%)	84,909 (41.07%)	69,156 (41.56%)	292,344 (40.91%)
Age, yr					
18-40	1,613 (1.18%)	2,288 (1.12%)	3,132 (1.51%)	2,055 (1.23%)	9,088 (1.27%)
41-50	5,599 (4.08%)	8,519 (4.17%)	9,634 (4.66%)	6,426 (3.86%)	30,178 (4.22%)
51-60	26,985 (19.66%)	38,095 (18.66%)	39,579 (19.14%)	29,131 (17.51%)	133,790 (18.72%)
61-70	50,606 (36.87%)	77,275 (37.86%)	75,643 (36.59%)	61,348 (36.87%)	264,872 (37.07%)
71-80	41,667 (30.36%)	62,569 (30.65%)	64,220 (31.06%)	53,927 (32.41%)	222,383 (31.12%)
81+	10,789 (7.86%)	15,363 (7.53%)	14,549 (7.04%)	13,518 (8.12%)	54,219 (7.59%)
Procedure type					
THA	57,438 (41.85%)	80,189 (39.29%)	85,856 (41.53%)	72,784 (43.74%)	296,267 (41.46%)
TKA	79,821 (58.15%)	123,920 (60.71%)	120,901 (58.47%)	93,621 (56.26%)	418,263 (58.54%)
Procedure year					
2018	56,128 (40.89%)	86,987 (42.62%)	80,638 (39.00%)	75,903 (45.61%)	299,656 (41.94%)
2019	51,081 (37.22%)	78,973 (38.69%)	77,060 (37.27%)	60,763 (36.53%)	267,877 (37.49%)
2020	30,050 (21.89%)	38,149 (18.69%)	49,059 (23.73%)	29,739 (17.87%)	146,997 (20.57%)
CCI					
mean ± SD	0.48 ± 1.06	0.50 ± 1.10	0.44 ± 1.05	0.47 ± 1.06	0.47 ± 1.07
Complication within 90 days					
Yes	1,113 (0.81%)	1,324 (0.65%)	1,664 (0.80%)	3,046 (1.83%)	7,147 (1.00%)
No	136,146 (99.19%)	202,785 (99.35%)	205,093 (99.20%)	163,359 (98.17%)	707,383 (99.00%)
Revision surgery within 1 year					
Yes	891 (0.65%)	1,153 (0.74%)	1,263 (0.61%)	1,404 (0.84%)	5,071 (0.71%)
No	136,368 (99.35%)	202,956 (99.44%)	205,494 (99.39%)	165,001 (99.16%)	709,459 (99.29%)

CCI = Charlson Comorbidity Index, THA = total hip arthroplasty, TKA = total knee arthroplasty

a Values are given as number of patients with percentage in parentheses within each region, except for mean CCI score.

**Figure 1:**
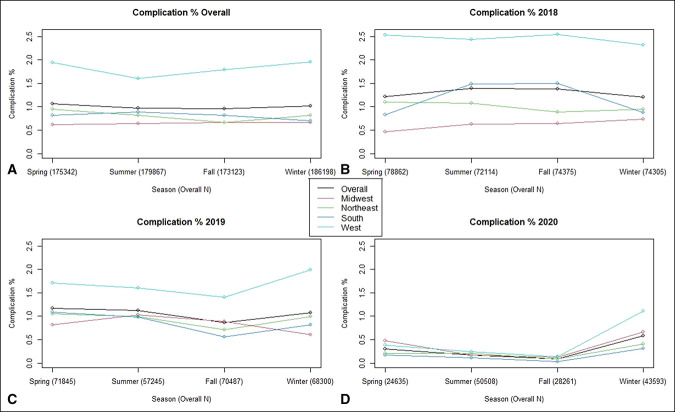
Graphs showing the complication rate for each region within each season. **A,** Overall complication rate from 2018 to 2020. **B,** Complication rate in 2018. **C,** Complication rate in 2019. **D,** Complication rate in 2020.

In each region, there were far fewer surgeries conducted in 2020 than in 2018 to 2019, likely due to elective surgery restrictions during the COVID-19 pandemic. Average CCI scores were similar across regions. Overall, the rate of complications dropped year to year, from 1.31% in 2018 to 1.08% in 2019 and finally to 0.30% in 2020 (Figure 1). The West had the highest 90-day complication rate of 1.83%, compared with 0.81% for the Northeast, 0.80% for the South, and 0.65% for the Midwest. Taking all regions into account, the complication rates for spring, summer, fall, and winter were 1.07%, 0.97%, 0.96%, and 1.01%, respectively (Figure 1).

**Figure 2:**
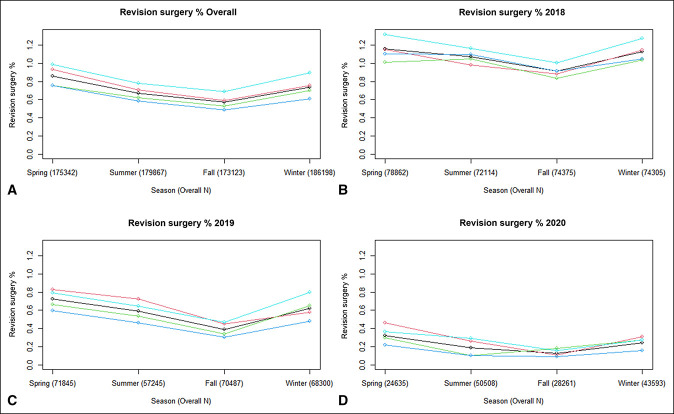
Graphs showing the revision surgery rate for each region within each season. **A,** Overall revision surgery rate from 2018 to 2020. **B,** Revision surgery rate in 2018. **C,** Revision surgery rate in 2019. **D**, Revision surgery rate in 2020.

The rate of revision surgeries followed a similar declining pattern year to year, from 1.06% in 2018 to 0.58% in 2019 and finally to 0.23% in 2020 (Figure 2). Although there was less disparity in the revision surgery rates region to region, the West again led with a revision surgery rate of 0.84% compared with 0.74% in the Midwest, 0.65% in the Northeast, and 0.61% in the South. The revision surgery rates for spring, summer, fall, and winter were 0.86%, 0.67%, 0.57%, and 0.74%, respectively (Figure 2).

### General Variation of Complication and Revision Surgery Risk

After Bonferroni and multivariable adjustment, there were no significant differences between racial groups in terms of 90-day complication risk, but female patients were found to have a significantly lower complication risk compared with males (adjusted odds ratio, aOR = 0.87, *P* < 0.001). Patients who underwent TKA had a lower risk of complication compared with those who underwent THA (aOR = 0.89, *P* < 0.001). Charlson Comorbidity Index increased the risk of complication (aOR = 1.17, *P* < 0.001), and there was also an increased risk of complication as patient age increased. Patients aged 81+ years had significantly increased complication rates compared with all younger patients except the group 18 to 40 years. Similar trends were seen for patients aged 71 to 80 years and 61 to 70 years.

In terms of 1-year revision surgery risk, multivariable logistic regression showed that Asian patients had a significantly lower risk of revision surgery within 1 year than African American patients (adjusted odds ratio, aOR = 0.55, *P* = 0.006) and Caucasian patients (aOR = 0.56, *P* = 0.004). Female patients had a significantly lower risk of revision surgery than males (aOR = 0.87, *P* < 0.001). Patients who underwent TKA had a higher risk of revision surgery than those who underwent THA (aOR = 1.39, *P* < 0.001). Those with higher CCI had a higher risk of revision surgery (aOR = 1.14, *P* < 0.001), and patients in age groups 71 to 80 and 80+ years had a significantly increased risk of revision surgery compared with all other age groups.

### Region, Season, and Regional-Seasonal Interaction

**Figure 3:**
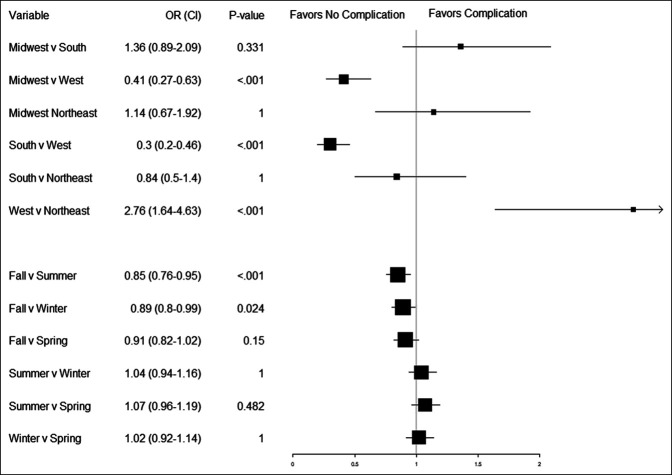
Forest plot showing odds ratios for LSMeans comparisons of complication risk for seasons and regions. For each factor, the odds ratio of the season/region to the left of the “v” is reported compared with the same season/region located to the right of the “v”. *P* values and 95% confidence intervals are determined through Bonferroni adjustment for multiple comparisons.

The analysis of complication risk yielded several significant regional and seasonal interactions. As seen in Figure 3, the West had a significantly higher risk of complication than the Northeast (aOR = 2.76, *P* < 0.001), the Midwest (aOR = 2.44, *P* < 0.001), and the South (aOR = 3.33, *P* < 0.001). This trend remained consistent across each season. Patients who underwent their primary TJA in the fall had a significantly lower risk of complication than in the summer (aOR = 0.85, *P* < 0.001) or the winter (aOR = 0.89, *P* = 0.024).

Investigation of seasonal effects within region yielded less striking results, and the only region to demonstrate any significant variance was the Northeast. Within the Northeast, patients treated in the fall had significantly lower complication rates than the spring (aOR = 0.64, *P* = 0.003) and had lower, but not significantly lower, complication rates than the winter (aOR = 0.70, *P* = 0.078) or the summer (aOR = 0.74, *P* = 0.409). Bonferroni-adjusted LSMeans comparisons of regions within each season and seasons within each region are included in Supplemental Figures S1 and S2, http://links.lww.com/JG9/A248 and http://links.lww.com/JG9/A249, respectively.

**Figure 4:**
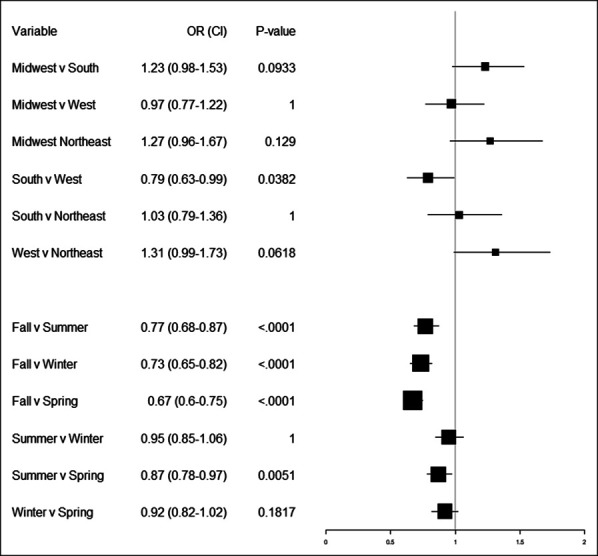
Forest plot showing odds ratios for LSMeans comparisons of revision surgery risk for seasons and regions. For each factor, the odds ratio of the season/region to the left of the “v” is reported compared with the same season/region located to the right of the “v”. *P* values and 95% confidence intervals are determined through Bonferroni adjustment for multiple comparisons.

Figure 4 displays the Bonferroni-adjusted LSMeans comparisons for revision surgery risk in each season and region pair. The South had a significantly reduced risk of revision surgery compared with the West (aOR = 0.79, *P* = 0.038), but no other significant differences were found in regional comparison when ignoring seasonal interactions. Patients treated in the fall had a significantly lower risk of revision surgery compared with those treated in the summer (aOR = 0.77, *P* < 0.001), the winter (aOR = 0.73, *P* < 0.001), and the spring (aOR = 0.87, *P* = 0.005).

No significant interaction effects were found between season and region in the model, and it appears that differences in revision surgery rates are determined marginally from seasonal and regional differences alone. Interaction analysis is displayed in Supplemental Figures S1 and S2, http://links.lww.com/JG9/A248 and http://links.lww.com/JG9/A249, respectively.

## Discussion

To our knowledge, this is the first study to evaluate regional variation in complication and revision surgery rates and the first to evaluate seasonal variation in revision surgery rates. We found that primary TJA patients in the West had a statistically higher risk of complications within 90 days of the index procedure compared with those in the Northeast, the Midwest, and the South. Revision surgery risk was determined to be relatively level across the geographic regions, but the South was found to have a significantly lower risk of revision surgery compared with the West. Additional analysis demonstrated that TJA procedures conducted in the fall had a lower risk of both complication and revision surgery than those conducted in the summer or the winter.

The literature supports the idea of seasonal variation in complication and revision surgery risk; however, most studies have focused on other types of surgery—such as strabismus correction or spinal fusion—^5-7^ and specific postoperative complications in isolation.^8‐10^ Within the AJRR, infection or inflammation was the number one reason for revision surgeries for both THA and TKA, accounting for 33% and 64% of revision surgeries of each procedure type, respectively. The risk for infection and other related postoperative complications have been shown to peak in the summer months,^8,9,11,12^ and weather may play a role in this variance. A recent single-center study found that individual risk for SSI increased by 1.3% for every degree increase in average temperature for the day of surgery.^13^ Similarly, Aghdassi et al^14^ found that patients who underwent surgery on days with temperatures of 20°C had an increased risk of SSIs compared with those who underwent surgery on days with temperatures of <5°C. Both results are in line with the results from our study, but it is important to note that neither of these prior works definitively determined a time of year when complications are at their lowest. As such, our findings with regard to fall procedures having a lower risk of both complication and revision surgery are of particular interest. The seasonal variance in revision surgery risk demonstrated in this study could also be correlated with these climate-induced differences in postoperative complications and infections, but additional study is necessary to further clarify this link.

Another explanation behind this seasonal variance in complication and revision surgery rates could be the July effect, which supposes that the influx of medical students into residency programs in the summer months leads to an increase in complications and morbidity during the transition period. This has been studied at some length with regard to seasonality of complications; however, evidence for the July effect is still heavily contested. In the orthopaedic literature specifically, reports largely suggest a lack of any such effect, and studies that did show increases in complications during the month of July found them to be the result of data from nonteaching hospitals.^15,16^ The data gathered by the AJRR are collected from a combination of both teaching and nonteaching hospitals, and overall, it seems improbable that the July effect could explain the trends found in our study, especially given that no statistical difference was found between summer and winter or summer and spring complication and revision surgery risk.

Our study also looked at regional data as an important factor for variance in complication and revision surgery rates. There are many different variables that could have affected these rates from region to region. Surgical volume in particular has been shown to be a strong indicator for complication and revision surgery risk,^17‐19^ and Lopez et al^20^ recently found that the rate of utilization of TJA is lowest in the West and highest in the South. It is possible that data from the West were drawn from surgical centers that individually saw lower surgical volumes. However, efforts were made to control for random effects such as hospital and surgeon factors in the analysis for this study, and it does seem unlikely that this bias would be inherent only to the singular region.

There is mixed evidence in orthopaedic studies about region as a risk factor for revision surgery. Some studies have shown no significant variation by region with regard to revision surgery due to periprosthetic joint infections or postoperative infections,^19,21^ but these studies have been limited by their focus on just a singular reason for revision surgery and not revision surgery as a whole. Curtin et al examined revision after TKA in the Medicare population and also found no significant variation by region.^17^ However, their study excluded all patients younger than 65 years, and this limited them to a much smaller patient population than that which was included in the current study. Other studies have shown that the South has the highest rate of revision for both total hip and knee arthroplasty,^22‐24^ a result that our data do not corroborate. These regional variances could be a result of differences in the level of activity across the regions or even ethnic geographic differences, but the real utility of this regional data is beyond just geographic appreciation.

The increased granularity achieved by including regional data allows for better appreciation of seasonal variation in complication and revision surgery rates and vice versa. Given the different climatologic patterns present in the four census regions of the United States, simply looking at seasonal or regional variation on their own fails to capture the broader trends. We found this to be of particular significance in the Northeast region where TJA procedures conducted in the fall demonstrated a significantly lower risk of complication compared with those in the spring and showed a lower, but not significantly lower, risk of complication compared with those in the winter and summer. Future study should focus on further characterizing these regional-seasonal combined variances, especially because the current data set does not allow for a definitive explanation as to why the regional variance exists.

Although the data presented are novel, there are several limitations in this study. First, this was a retrospective, observational study and was prone to the limitations inherent in such study design. The data for this study were obtained from the AJRR, which is an administrative data set, and our conclusions are dependent on the reliable coding and documentation of revision surgeries after TJA. In addition, although the AJRR includes data from 1451 different facilities across all regions of the United States, around 3500 hospitals perform TJA in the United States,^25^ yielding a capture rate of approximately only 41%. The findings in this study assume that both primary operation and subsequent complications were tracked within an institution that submits data to the AJRR, although this may not always have held true. There is a similar assumption for revision surgeries within this data set, and as such, the complication and revision surgery rates and risks calculated from the data may be underestimated. The database also does not allow for easy determination of the exact reason for revision surgery, which makes it difficult to accurately pinpoint the sources of the overlying trends. Finally, the data we obtained from the AJRR only included three years, and although this yielded a substantial number of patients, a more prolonged period to assess these trends, especially seasonality, might be warranted.

Despite these limitations, our work demonstrates definitive effects of region and season on complication and revision surgery rates and underlines the need for further study to characterize the effects of region and season as risk factors for these outcomes for other elective orthopaedic procedures. Investigating the reasons behind these variations should also be a priority to reduce the revision surgery burden on the healthcare system and patients alike.

## Conclusion

This retrospective study analyzed the effects of region, season, or their interaction on the risk of complications within 90 days and the risk of revision surgery within 1 year of the index TJA. Multivariable logistic regression demonstrated a significantly increased risk of complication in the West compared with any of the other regions (Northeast, Midwest, and South) and a significantly increased risk of revision surgery in the West compared with the South. Seasonally, fall TJA operations demonstrated a lower risk of both complications and revision surgeries compared with operations conducted in the summer or winter. Further study should focus on isolation of individual factors for these overlying variations.
